# Evaluation of short-TE ^1^H MRSI for quantification of metabolites in the prostate

**DOI:** 10.1002/nbm.3082

**Published:** 2014-02-12

**Authors:** Meer Basharat, Maysam Jafar, Nandita M deSouza, Geoffrey S Payne

**Affiliations:** 1 CRUK and EPSRC Cancer Imaging Centre, Institute of Cancer Research, Royal Marsden NHS Foundation TrustSutton, Surrey, UK

**Keywords:** prostate, spectroscopy, citrate, spermine

## Abstract

Back-to-back ^1^H MRSI scans, using an endorectal and phased-array coil combination, were performed on 18 low-risk patients with prostate cancer at 3 T, employing TEs of 32 and 100 ms in order to compare metabolite visualization at each TE. Outer-volume suppression of lipid signals was performed using regional saturation (REST) slabs and the quantification of spectra at both TEs was achieved with the quantitation using quantum estimation (QUEST) routine. Metabolite nulling experiments in an additional five patients found that there were negligible macromolecule background signals in prostate spectra at TE = 32 ms. Metabolite visibility was judged using the criterion Cramér–Rao lower bound (CRLB)/amplitude < 20%, and metabolite concentrations were corrected for relaxation effects and referenced to the data acquired in corresponding water-unsuppressed MRSI scans. For the first time, the prostate metabolites spermine and myo-inositol were quantified individually *in vivo*, together with citrate, choline and creatine. All five metabolite visibilities were higher in TE = 32 ms MRSI than in TE = 100 ms MRSI. At TE = 32 ms, citrate was visible in 99.0% of lipid-free spectra, whereas, at TE = 100 ms, no metabolite simulation of citrate matched the *in vivo* peaks. Spermine, choline and creatine were visualised separately in 30.4% more spectra at TE = 32 ms than at TE = 100 ms, and myo-inositol in 72.5% more spectra. *T*_2_ values were calculated for spermine (53 ± 16 ms), choline (62 ± 17 ms) and myo-inositol (90 ± 48 ms). Data from the TE = 32 ms spectra showed that the concentrations of citrate and spermine secretions were positively correlated in both the peripheral zone and central gland (*R*^2^ = 0.73 and *R*^2^ = 0.43, respectively), and that the citrate content was significantly higher in the former at 64 ± 22 mm than in the latter at 32 ± 16 mm (*p* = 0.01). However, lipid contamination at TE = 32 ms was substantial; therefore, to make clinical use of the greater visualisation of prostate metabolites at TE = 32 ms rather than at TE = 100 ms, three-dimensional MRSI at TE = 32 ms with effective lipid suppression must be implemented.

## INTRODUCTION

The management of prostate cancer depends on the correct staging and grading of prostate tumours. Prostate cancer is a highly heterogeneous disease and therefore treatment should be specific for each case. Staging is performed with information from morphological imaging techniques, such as diffusion-weighted MRI (DW MRI) 1, whereas grading requires histological evaluation of tissue, as structural changes do not always reflect tumour grade 2. A 12-core prostate biopsy is the current clinical standard for the grading of tumours, but this method has poor specificity because of the variable nature of random sampling of small fractions of the prostate 3. Alternatively, MRSI is capable of measuring non-invasively the metabolic content of all tissues. In prostate tumours, the choline content increases 4 and the citrate level is reduced 5; these changes have been shown to relate to tumour grade 6, and therefore MRSI has the potential to provide a virtual biopsy of the entire prostate.

The most commonly used pulse sequence for ^1^H MRSI in the prostate is point-resolved spectroscopy (PRESS), 90° — *τ* — 180° — ½TE — 180° — (½TE – *τ*) — *acquire*, with a total TE of more than 130 ms 7. The use of such long TEs allows water and lipid suppression pulses to be accommodated into the sequence and enables short-*T*_2_ lipid signals to decay. However, in prostate spectra, it is often difficult to visualise the choline peak at 3.21 ppm separately from the polyamine and creatine peaks at 3.10 and 3.03 ppm, respectively. Instead, a composite metabolite area is usually measured and the metabolite ratio (choline + polyamines + creatine)/citrate is reported 8. *In vivo* prostate MRSI studies usually report the metabolite ratio, but this has shown a large overlap of values between tumours of different grades, possibly as a result of effects from the underlying polyamine peaks; polyamines are known to be reduced in prostate tumours 9. One study used a three-tier scoring method to classify polyamine levels in spectra (undetectable, lower than choline peak, higher than choline peak) 10, whereas absolute citrate and choline concentrations have been calculated in other studies 11–13. However, no study has yet reported absolute polyamine concentrations *in vivo*.

Despite its potential for the grading of tumours, MRSI is not currently used in clinical examinations of the prostate. The reasons for this include the poor intrinsic metabolite signal, lipid contamination from periprostatic fat tissues, unclear optimal scanning parameters and the requirement for additional time and expertise to manually process and interpret the data. The purpose of this study was to assess short-TE (TE = 32 ms) MRSI for the identification of individual metabolite signals in the prostate. Short-TE MRSI should offer improved signals over longer TE MRSI, as there is reduced time for transverse relaxation and *J* modulation of signals to occur. Using this method, we calculated the absolute concentrations of citrate, spermine (the main prostate polyamine), choline, creatine and myo-inositol for the peripheral zone (PZ) and central gland (CG). We also compared metabolite visibilities at this TE with those at TE = 100 ms, the TE conventionally used at our centre.

## SUBJECTS AND METHODS

### Patients and imaging methods

Eighteen low-risk patients with prostate cancer (aged 46–77 years), managed by active surveillance, underwent MRI examination under a protocol approved by our institutional research ethics committee. All studies were performed on an Achieva 3-T scanner (Philips Healthcare, Best, the Netherlands) using an endorectal coil (MEDRAD, Indianola, PA, USA) combined with a phased-array coil. The endorectal coil was filled with 60 mL of perfluorocarbon for body tissue susceptibility matching 14. A turbo spin-echo sequence was employed to acquire *T*_2_-weighted images in the transverse plane using the following parameters: TR/TE = 3643/110 ms; four averages; matrix size, 220 × 184; slice thickness, 2.2 mm; slice separation, 0.1 mm; right–left field of view, 140 mm.

### Spectroscopic imaging methods

Two-dimensional (2D) MRSI was performed using a PRESS-localised volume. The MRSI grid and PRESS volume were planned using the transverse *T*_2_-weighted MR images and placed through the largest part of the prostate in the superior–inferior direction. Each PRESS volume had a thickness of 10 mm and was planned so as to include the maximum volume of the prostate possible in the transverse plane, whilst excluding periprostatic fat. As a result of the shortness of the TE = 32 ms PRESS sequence, no lipid suppression pulses could be inserted, and so were not added to MRSI at either TE. Outer-volume lipid suppression was performed using 60-mm regional saturation (REST) slabs; four REST slabs were used on the first 10 patients but, for the last 11 patients, two extra REST slabs were added to further reduce lipid contamination.

Each 2D MRSI scan was performed first with water suppression, with the frequency offset centred at citrate, and then without water suppression, with the frequency offset centred at water. This was performed in order to obtain an unsuppressed water signal for metabolite concentration referencing. Water suppression was achieved by tipping the bulk water magnetisation at the outset by just over 90°, so that the longitudinal magnetisation was nulled at *t* = 0. Spoiler gradients were then applied in order to remove the water's transverse magnetisation. Each 2D MRSI scan with water referencing lasted 6 min.

Two consecutive 2D MRSI examinations with *τ* = 8.86 ms, TE = 32.00 ms and *τ* = 8.86 ms, TE = 100.00 ms were performed on all patients. Receiver gain was manually set to 0 dB on all scans, so that signals at both TEs could be compared directly. The amplitude-modulated radiofrequency pulses had the following attributes: 90° pulse length, 7.1 ms; bandwidth, 1987 Hz; 180° pulse length, 6.9 ms; bandwidth, 1263 Hz. Other acquisition parameters were as follows: TR = 1410 ms; 2048 time domain points; bandwidth, 2000 Hz; Number of Sample Averages (NSA) = 1; field of view, 120 mm × 120 mm × 10 mm; 12 × 12 phase encoding grid. Together with the additional ∼2–3 min required for set-up, the total MRSI scanning time was ∼14–15 min for each patient.

### Voxel classification and spectral processing

After inspecting all relevant *T*_2_-weighted MR images, prostate voxels were identified as those in which prostate tissues constituted greater than 50% of the non-lipid tissues within the voxel. These voxels were further classified according to the dominant prostate zone tissue type within the voxel: PZ or CG. For PZ voxels, the percentage of the voxel which contained PZ tissue was also recorded.

Spectral data were processed offline using jMRUI v4.0 (www.mrui.uab.es/mrui/). All spectra were zero filled and the Hankel–Lanczos singular value decomposition (HLSVD) filter was used to remove residual water and lipid signals above 4.1 ppm and below 1.65 ppm, respectively. After filtering, any spectra still containing negative excursions from the overall baseline (as a result of lipid distortion) were excluded.

### Metabolite nulling methods

Correct spectral fitting depends on the identification of contributions from broad resonances from macromolecules; these contributions have not been investigated previously in the prostate. To test for the presence of such components in TE = 32 ms spectra, the method of metabolite nulling was used, as applied previously in the brain 15. An inversion pulse was applied at a time (inversion time, TI) before the localisation sequence began. Short-*T*_1_ macromolecule signals recover to near-equilibrium by time *t* = 0, whereas long-*T*_1_ metabolite signals are approximately nulled.

Five additional patients with prostate cancer were examined with single-voxel spectroscopy, back to back, with and without metabolite nulling. Voxels were placed to include regions of both PZ and CG; the voxel size was 15 mm × 15 mm × 20 mm, the inversion bandwidth was 1500 Hz for metabolite nulling and the other parameters were the same as in the TE = 32 ms MRSI scans. Experiments on the first two subjects used TIs ranging from 250 to 750 ms, and indicated that TI = 300 ms was the optimal choice with which to null prostate metabolite signals. Another three subjects were then scanned using TI = 300 ms.

### Metabolite basis sets

Basis sets for three of the main prostate metabolites (choline, creatine and myo-inositol) were simulated with NMR Scope (part of the jMRUI package) using chemical shift values and *J*-coupling constants found in the literature 16. Choline: Spin 1, δ = 3.185 ppm, *n* = 9; Spin 2 and 3, δ = 4.054 ppm, *n* = 1; Spin 4 and 5, δ = 3.501 ppm, *n* = 1, *J*_24_ = 3.140 Hz, *J*_25_ = 6.979 Hz, *J*_34_ = 7.011 Hz, *J*_35_ = 3.168 Hz. Creatine: Spin 1, δ = 3.027 ppm, *n* = 3; Spin 2, δ = 3.913 ppm, *n* = 2. Myo-inositol: Spin 1 and 3, δ = 3.532 ppm, *n* = 1; Spin 2, δ = 4.054 ppm, *n* = 1; Spin 4 and 6, δ = 3.614 ppm, *n* = 1; Spin 5, δ = 3.269 ppm, *n* = 1, *J*_12_ = 2.889 Hz, *J*_16_ = 9.938 Hz, *J*_23_ = 3.006 Hz, *J*_34_ = 9.997 Hz, *J*_45_ = 9.485 Hz, *J*_56_ = 9.482 Hz. The chemical shift difference Δ*ν* and the geminal *J*-coupling constant *J*_AB_ used to simulate the basis set of the citrate AB spin system were extracted directly from the clinical data. All four of these basis sets were simulated using hard radiofrequency pulses and timing parameters from the two PRESS sequences. Spermine [at 71% by content, the predominant polyamine 17] contains 20 spins with 12 vicinal *J* couplings, and therefore was too complex to be simulated by NMR Scope. To obtain a spermine basis set, an experimental spermine spectrum was acquired from a phantom containing 20 mm spermine at pH 7.6, using the clinical 2D MRSI scan with only the phased-array coil. Three singlet basis sets were also simulated at 2.10, 3.72 and 4.10 ppm in order to fit the resonances of other overlapping spin species: lipids (2.10 ppm), the minor polyamines spermidine and putrescine (2.10 ppm), glutamate and glutamine (2.10 and 3.72 ppm) and lactate (4.10 ppm) 18. The amplitudes of these peaks are not reported as the resonances have a composite metabolic origin.

### Quantification of metabolites and metabolite visibility

Metabolites in the spectra were fitted and quantified with the quantitation using quantum estimation (QUEST) procedure in jMRUI 19. QUEST is a semi-parametric time-domain metabolite-fitting approach which fits spectra using metabolite free induction decay (FID) models (basis sets), the parametric part, and a background handling routine, the non-parametric part. After fitting, QUEST returns an amplitude and Cramér–Rao lower bound (CRLB) for each metabolite. CRLB is the minimum possible uncertainty in fitting the metabolite (a lower bound), given the quality of the data and the validity of the fitting model. (Lower CRLBs are therefore suggestive of improved metabolite visibility in the data, although true uncertainty is always ≥ CRLB.) In this study, we used CRLB/amplitude < 20% as the criterion to discriminate well-fitted metabolites from more poorly fitted metabolites, providing an upper bound for metabolite visibility.

The *Subtract* approach was used for background handling as this has already shown better bias–uncertainty trade-off in metabolite fitting than the other available option, *InBase* 20. First, *Subtract* truncates the FID from *t* = 0 by a user-defined number of points, and the remaining FID is fitted using the metabolite FID basis sets. This metabolite fit is then subtracted from the original non-truncated FID, leaving a signal which is the macromolecule background signal alone. This routine is valid as long as the true macromolecule signal decays fully during the truncation period. The amount of truncation used is therefore critical and, in this study, this amount was decided after consideration of the metabolite-nulled data.

For each spectrum, the frequency positions and linewidths of every individual metabolite and singlet resonance were independently and automatically adjusted from their original simulation values to fit the data; linewidth broadening was constrained between 0 and 11.1 Hz and frequency shifting was constrained between −0.03 and +0.03 ppm. *T*_2_ values were calculated from the metabolite amplitudes at TE = 32 ms and TE = 100 ms, for metabolites with acceptable fits at both TEs. Absolute metabolite concentrations were produced from the TE = 32 ms spectra by referencing metabolite amplitudes to the magnitude of the raw water FID (from the matching water-unsuppressed spectrum) and using an assumed water tissue concentration of 46.1 m 21. *T*_1_ and *T*_2_ corrections were made to the concentration results from QUEST using *T*_1_ and *T*_2_ values from the literature or, where available, from the data.

## RESULTS

In the 18 patients, 310 prostate voxels were identified [109 PZ, 189 CG, five mixed (equal amounts of PZ tissue and non-PZ tissue) and seven suspected tumour] based on the appearance of the *T*_2_-weighted and DW MRI information, as assessed by an experienced observer.

### Assessment of short- and long-TE spectra

**Figure 1:**
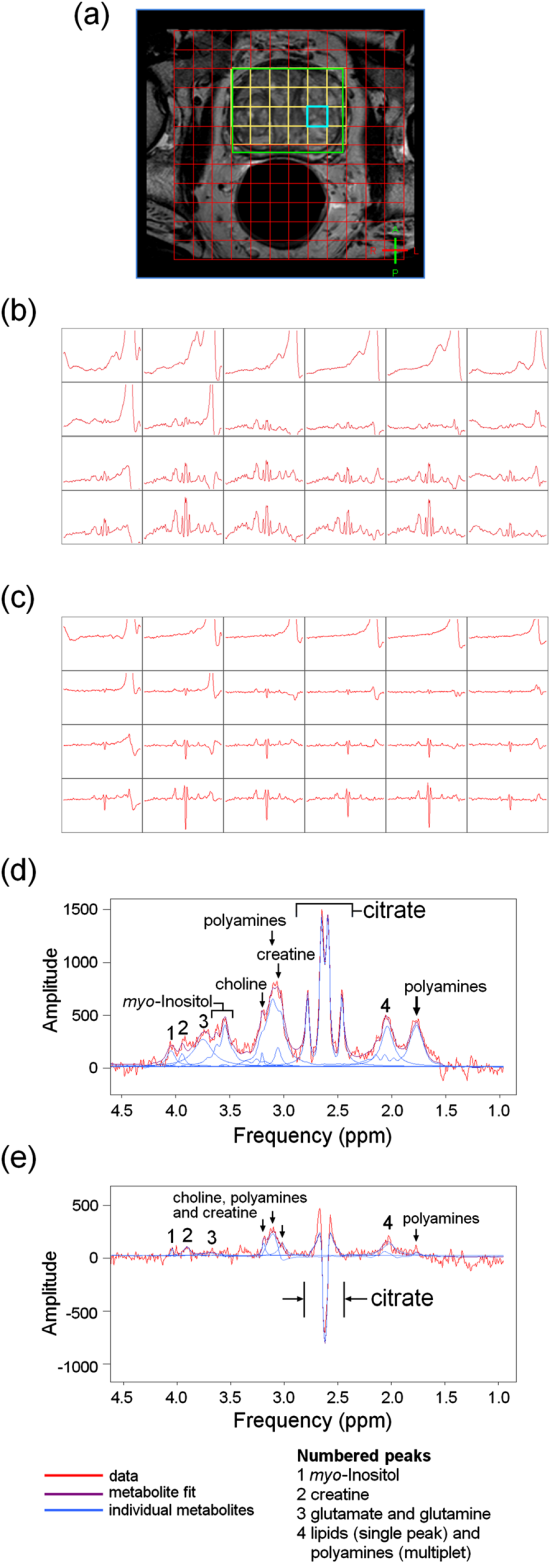
Transverse *T*_2_-weighted MR image of a prostate (a) showing point-resolved spectroscopy (PRESS) volume (green) and spectroscopic grid placement (red). Raw spectral grids are shown for TE = 32 ms MRSI (b) and TE = 100 ms MRSI (c) from 4.5 to 1.0 ppm on the same vertical scale as each other. Processed spectra from the prostate voxel highlighted in (a) are shown for TE = 32 ms (d) and TE = 100 ms (e) on the same vertical scale as each other.

Example TE = 32 ms and TE = 100 ms spectra from a prostate voxel are shown in Figure 1. Several additional metabolite peaks were noticed in TE = 32 ms prostate spectra compared with the corresponding TE = 100 ms spectra, notably myo-inositol at 3.55 and 3.62 ppm, glutamate and glutamine at 3.75 ppm and polyamine resonances at 1.80 ppm. Metabolite visibilities are reported in Table 1.

**Table 1:** Metabolite visibility. Percentage of voxels that have metabolite Cramér–Rao lower bound (CRLB)/amplitude < 20% (an upper bound for metabolite visibility) using spectra from all voxels (top rows) and voxels that are lipid free at both TEs (bottom rows), also subdivided into visibilities in both prostate zones

All voxels	Citrate	Spermine, choline and creatine	Spermine	Choline	Creatine	Myo-inositol
TE = 32 ms	58%	27%	38%	32%	32%	44%
PZ	58%	23%	34%	25%	31%	35%
CG	59%	29%	40%	36%	34%	49%
TE = 100 ms	–	30%	53%	57%	43%	2%
PZ	–	28%	53%	53%	46%	0%
CG	–	30%	51%	57%	40%	3%
Lipid-free voxels						
TE = 32 ms	99%	66%	94%	79%	79%	75%
PZ	100%	67%	98%	71%	88%	62%
CG	98%	67%	92%	82%	77%	81%
TE = 100 ms	–	35%	62%	67%	51%	2%
PZ	–	35%	65%	66%	58%	0%
CG	–	36%	61%	67%	47%	4%

CG, central gland; PZ, peripheral zone.

Metabolite visibility decreased with distance from the endorectal coil because of signal drop-off. Of the 52 lipid-free TE = 32 ms spectra in which the CRLB/amplitude was greater than 20% for any metabolite, 41 spectra originated from voxels more than 10 mm away from the endorectal coil. Citrate visibility in voxels adjacent to the endorectal coil was lower than that in all other voxels, across all patients (CRLB/amplitude = 1.46% *versus* 2.22%, *p* = 0.8%).

### Quantitative assessment: lipid contamination

Spectral distortion from large lipid signals caused a compromise in quantification of all metabolites in 41.2% of spectra at TE = 32 ms and 7.9% of spectra at TE = 100 ms. Smaller lipid distortions still affected the quantification of the 1.8-ppm polyamine peak which, in turn, compromised the quantification of the choline and creatine resonances; altogether, lipids affected the quantification of spermine, choline and creatine in 59.4% of spectra at TE = 32 ms and 12.0% of spectra at TE = 100 ms.

### Quantitative assessment: background macromolecule signals

**Figure 2:**
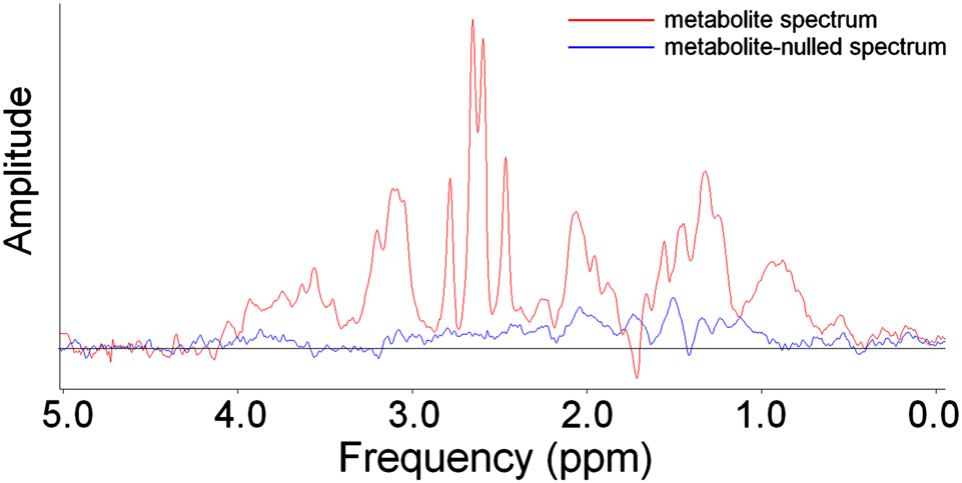
Consecutive *in vivo* TE = 32 ms prostate spectra (after removal of residual water peaks) without and with metabolite nulling.

In the four metabolite-nulled spectra using TI = 300 ms, there were no observable signals, except for an inverted choline peak in one. Total areas under the metabolite-nulled spectra between 1.7 and 4.5 ppm in the other three cases were 2.8%, 5.5% and 8.3% (Fig. 2) of the area in the corresponding metabolite spectra. Because of this lack of significant broad peaks from macromolecules in short-TE spectra, we used the minimum possible background handling in QUEST: only one FID point was truncated in the *Subtract* routine.

### Qualitative assessment: fitting the citrate resonance

**Figure 3:**
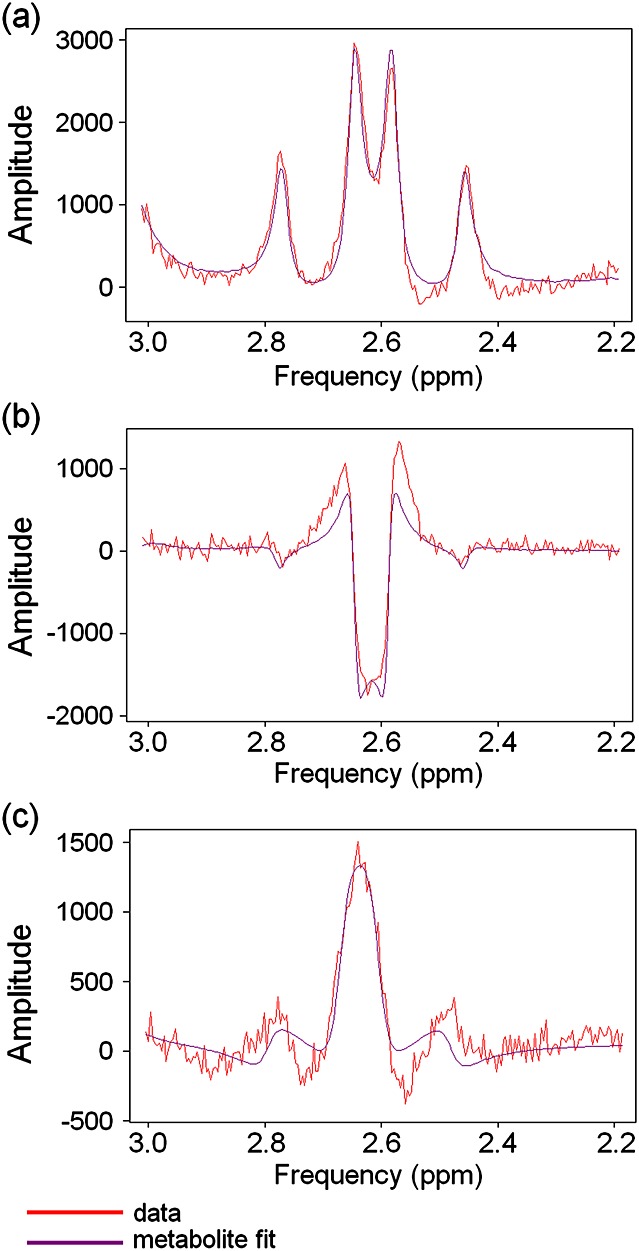
Example citrate basis set fits to *in vivo* prostate spectra: (a) TE = 32 ms spectrum fitted using Δ*ν* = 18.21 Hz and *J*_AB_ = 15.25 Hz [Σ(residue^2^)/Σ(noise^2^) = 7.10] shows good matching to all four citrate peaks; (b) TE = 100 ms spectrum from the same voxel fitted using Δ*ν* = 17.32 Hz and *J*_AB_ = 16.25 Hz [Σ(residue^2^)/Σ(noise^2^) = 10.43] shows mismatch around the positive inner lobes between the data and the simulation; (c) an examination using TE = 145 ms and *τ* = 25 ms was performed on a patient, solely for the purpose of this figure; this spectrum fitted using Δ*ν* = 18.00 Hz, *J*_AB_ = 16.25 Hz, *τ* = 25 ms and TE = 145 ms [Σ(residue^2^)/Σ(noise^2^) = 4.89] shows a mismatch between the data and the simulation around the outer sidebands.

At TE = 32 ms, the four *J*-modulated citrate peaks were positive in amplitude and displayed a roofing effect (Fig. 3a). The frequencies of the four peaks were used to calculate Δ*ν* and *J*_AB_ for the citrate simulation. Δ*ν* and *J*_AB_ varied by <2 Hz across all spectra but, for efficiency, only one basis set which matched the most spectra was used for quantification (Δ*ν* = 18.21 Hz and *J*_AB_ = 15.25 Hz). Quantification using one common basis set was highly efficient, and errors caused by basis set inaccuracies (±1 Hz) were expected to be negligible. At TE = 100 ms, however, no set of Δ*ν* and *J*_AB_ values was found whose simulation fitted all three characteristics of the *in vivo* citrate spectral shape: large inverted central peaks with smaller positive inner lobes and outer dispersive sidebands. Instead, a compromise was reached by using a basis set which matched the large inverted central peaks and the smaller sidebands, but not the positive inner lobes (Δ*ν* = 17.32 Hz and *J*_AB_ = 16.25 Hz, Fig. 3b). Any amplitudes, *T*_2_ values or CRLBs calculated using this basis set are underestimates because the fitting model is incorrect. Although this basis set inclusion is unsatisfactory for the fitting of citrate, if excluded, the other metabolite peaks would be distorted in an attempt to fit citrate 22.

**Figure 4:**
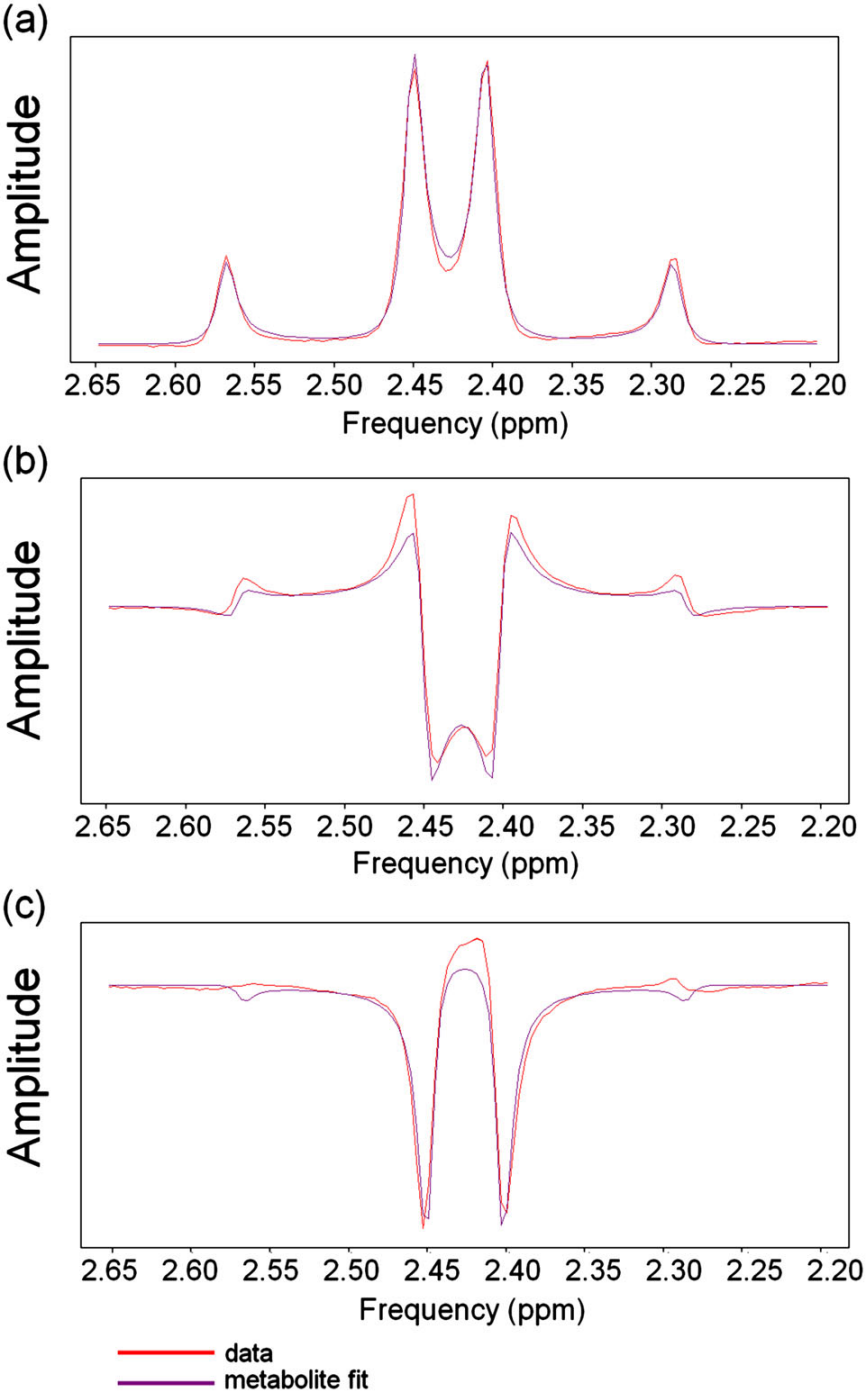
MRSI spectra from a phantom containing 20 mm citrate: (a) TE = 30 ms; (b) TE = 100 ms; (c) TE = 200 ms; all fitted using Δ*ν* = 14.45 Hz and *J*_AB_ = 15.10 Hz. There is excellent agreement between the shape of the data and the simulation for TE = 30 ms, but not for the longer TEs.

TE = 145 ms is commonly used for 3-T prostate MRSI 13,23 (we used TE = 100 ms as the central citrate peaks have a large, negative amplitude, but suffer from less *T*_2_ decay). Therefore, we performed PRESS on two additional patients using TR/TE = 1410/145 ms. Citrate simulations were also inexact for this TE (Fig. 3c), as well as for TE ≥ 100 ms spectra from a citrate phantom (Fig. 4).

### Quantitative assessment: metabolite *T*_2_ values and absolute concentrations

**Figure 5:**
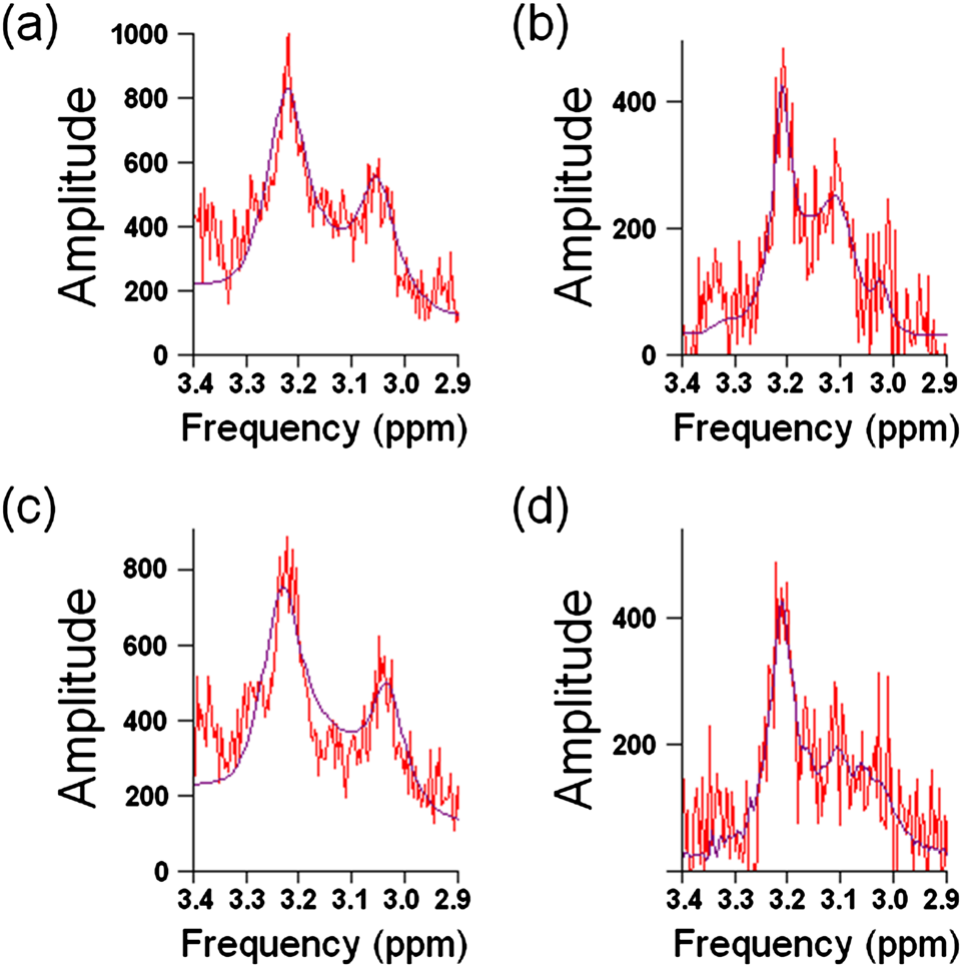
Example prostate spectra from one patient in which choline was judged to be fitted with little overlap of the other metabolites. The TE = 32 ms spectrum (a) and TE = 100 ms spectrum (b) originate from the same voxel. The TE = 32 ms spectrum (c) and TE = 100 ms spectrum (d) originate from a second voxel. No apodisation has been applied.

*T*_2_ values were calculated for spectra with metabolite CRLB/amplitude < 20% and where the metabolite fit was judged to be acceptable at both TEs. This was necessary because of the ambiguity of metabolite fits; *T*_2_ values were only calculated from spectra in which metabolite separation was distinct (Fig. 5), which was only the case for spermine, choline and myo-inositol (see Table 2).

**Table 2:** Relaxation time constants used to correct metabolite concentrations, either from the data (mean ± standard deviation) or from references where shown

Metabolite	Citrate	Spermine	Choline	Creatine	Myo-inositol	Water
*T*_1_ (ms)	470 ± 140 24	1025 25	1100 ± 400 24	1375 26	997 26	1597 ± 42 28
*T*_2_ (ms)	170 ± 50 24	53 ± 16 (*n* = 26)	62 ± 17 (*n* = 11)	209 ± 97 27	90 ± 48 (*n* = 7)	71 ± 10 (CG), 114 ± 30 (PZ) 12

CG, central gland; PZ, peripheral zone.

**Figure 6:**
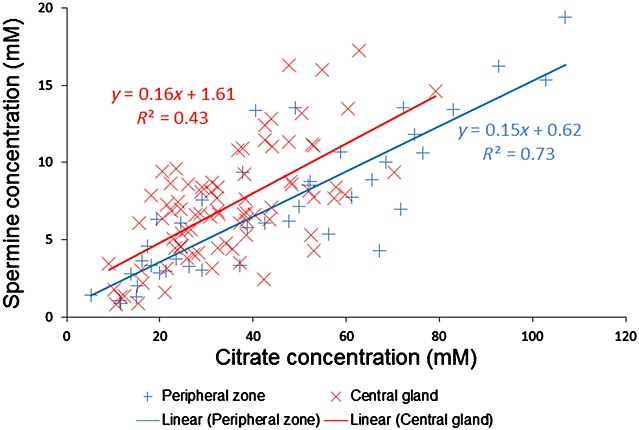
Correlation of citrate and spermine absolute concentrations by prostate region from TE = 32 ms spectra across all patients.

Metabolite concentrations from the TE = 32 ms spectra from all metabolite fits with CRLB/amplitude < 20% were relaxation corrected using the relaxation constants from this study or from the literature (Table 2). Table 3 shows the final *T*_1_- and *T*_2_-corrected absolute concentrations. Concentrations of citrate and spermine were found to be correlated (Fig. 6). A two-tailed test for the significant difference between the PZ and CG correlations returned *p* = 1.6%, showing that the concentrations are more tightly correlated in PZ.

**Table 3:** Absolute prostate metabolite concentrations from TE = 32 ms prostate spectra, *T*_2_ corrected and quantified employing quantitation using quantum estimation (QUEST)

Metabolite	Citrate	Spermine	Choline	Creatine	Myo-inositol
Central gland voxels					
Mean concentration ± standard deviation (mm)	32 ± 17	6.9 ± 3.7	4.9 ± 3.6	8.3 ± 7.4	14.6 ± 11.5
*n*	125	87	79	76	100
>75% peripheral zone voxels					
Mean concentration ± standard deviation (mm)	64 ± 22	10.0 ± 4.4	6.8 ± 3.2	8.9 ± 4.9	10.3 ± 7.6
*n*	16	13	10	13	14
*p* for difference between zones	0.01	0.07	0.36	0.41	0.06

## DISCUSSION

In this study, the metabolites spermine and myo-inositol have been quantified individually from one-dimensional spectra for the first time in the *in vivo* prostate, together with citrate, choline and creatine which have been measured previously. As known previously, we found that PZ was metabolically distinct from CG, producing double the amount of citrate (*p* = 0.01). Citrate and spermine secretions are signs of healthy prostate function, and our data demonstrate that citrate and spermine contents are positively correlated in both prostate zones, particularly in PZ, where glandular structure and secretory function predominate. This correlation is in agreement with previous high-resolution magic angle spinning studies of prostate tissue samples (*R*^2^ = 0.88) 29 and of prostatic fluid samples 30. Myo-inositol was abundant in PZ (10.3 ± 7.6 mm), which is to be expected from previous prostate extract studies 30,31, although both of these studies present a large variation in concentration and conflicting changes in concentration between normal and tumour tissue.

Absolute quantification of metabolites was an important goal in this study. The average concentrations found in this study for citrate, choline and creatine in PZ were 20–100% higher than literature values. Although there is likely to be a large natural variation in metabolite concentrations over the 20 patients, several other factors can affect the calculation of absolute metabolite concentrations. The significance of broad underlying peaks from macromolecules or immobile water molecules (with *T*_2_ < < 10 ms) has been identified in brain spectra 15, but their significance in prostate spectra has not been established previously. We investigated the presence of macromolecule signals in (TE = 32 ms) prostate spectra for the first time and, unlike in the brain, found that their size was negligible compared with the metabolite signals. Minimal background handling was therefore needed in the metabolite quantification. We did not infer metabolite *T*_1_ values from these metabolite nulling experiments as TR was shorter than *T*_1_ (TR = 1410 ms) and metabolite amplitudes were not measured over a TI series.

Second, the metabolite *T*_2_ values calculated in this study were derived from a two-point decay curve only, and were all shorter than values in the literature: spermine, 53 ± 16 ms *versus* 71 ms 25; choline, 62 ± 17 ms *versus* 220 ± 90 ms 24; myo-inositol, 90 ± 48 ms *versus* 197 ± 14 ms (brain) 32; creatine was poorly defined at TE = 100 ms, although creatine *T*_2_ = 209 ± 97 ms at 1.5 T 27. Our purpose in calculating *T*_2_ was simply to correct for amplitude loss over time. Visual inspection of the spectra revealed amplitude reductions of >50% for these metabolites between TE = 32 ms and TE = 100 ms (see Fig. 1d, e), suggesting short *T*_2_ values. Only spectra with no overlapping metabolites were chosen for *T*_2_ calculation and the metabolite nulling experiments exclude the presence of other broad underlying components. *J* modulation is also not sufficient to explain the signal loss of myo-inositol observed *in vivo* (only 77% reduction in simulations). These arguments provide support for the calculated *T*_2_ values. However, our *T*_1_ and *T*_2_ relaxation corrections were based on a mono-exponential model of decay, whereas water is known to have multi-exponential signal decay in the prostate 33. The investigation of multi-exponential decay of metabolite signals *in vivo* was not a priority as we were concerned with simple signal decay between TE = 32 ms and TE = 100 ms, and such scans would have increased the examination time. However, multi-exponential decay could explain the disparity between the *T*_2_ values found in this study and other studies, in which different TE regimes were used.

Another factor affecting the calculated absolute concentrations is that *T*_1_ and *T*_2_ (used in the concentration correction) are expected to have a range of values *in vivo*. Using any single value for relaxation correction is therefore imperfect (although scans to determine individual *T*_1_ and *T*_2_ values would be lengthy). However, these variations will have far less effect on calculations made from short-TE metabolite amplitudes, such as those measured here, than from longer TE metabolite amplitudes. Water concentration variation is also possible, although previous work has indicated that water variation across the prostate is negligible 12. Although there is likely to be bound, invisible water in the prostate 34, the amount has never been quantified; therefore, we assumed that 100% of water was visible. However, other studies have also used different assumed water concentrations to calculate the absolute metabolite concentrations [e.g. 39 m 11, 55 m 13], and this will contribute to the disparity in values. The chemical shift artefact (CSA) is also important to consider, as 40% of lipid-free TE = 32 ms voxels were edge voxels. The PRESS volume widths (20–70 mm in-plane) give CSAs for the primary spermine, choline and creatine peaks of 1–4 mm and, for *myo*-inositol, 2–7 mm. Therefore, real concentrations may be higher than those calculated, owing to the reduction in resonance amplitudes caused by the edges of the PRESS box being shifted into these voxels. However, as most resonances are at a higher frequency than the citrate resonance, the CSA will only be in a single direction, thus only affecting half of the edge voxels, or 20% of the spectra overall.

The satisfactory quantification of citrate is important in prostate MRSI, as it is the largest signal in healthy prostate. In the past, citrate peaks have been integrated 35, but, strictly, without the information provided by a simulation or a reference spectrum, the area of *J*-modulated citrate peaks can only be used to compare amplitudes between spectra, and not to calculate absolute metabolite concentrations. The spectrum of citrate, a strongly *J*-coupled AB spin system, consists solely of *J*-coupled peaks. Therefore, the correct simulation of the citrate spectrum depends on the precise chemical shift difference Δ*ν* and geminal *J*-coupling constant *J*_AB_ of the citrate system (^4^ *J* couplings are negligible, ^4^ *J* < < 1 Hz), as well as the radiofrequency pulse sequence applied. Citrate Δ*ν* and *J*_AB_ varied by <2 Hz *in vivo*, but quantification errors as a result of the use of one basis set were expected to be negligible. In addition, tailored quantification for each spectrum would be prohibitively lengthy. However, the simulations of citrate at TE = 100 ms and TE = 145 ms did not match well the shape of either *in vivo* or phantom citrate spectra; at TE = 100 ms, the positive inner lobes of the simulated citrate spectrum were ∼50% of the *in vivo* amplitude (Fig. 3b). Simulations in programs other than NMR Scope have been used to fit citrate in other studies, but residues are always common. Further, previously published prostate spectra have originated from smaller voxels than in this study, and therefore have had insufficient signal-to-noise ratio to detect these fitting problems. We expect that there is a fundamental issue with the simulation of citrate, as other simulations using many combinations of Δ*ν* and *J*_AB_, as well as using software available more recently with Gaussian and sinc radiofrequency pulses, did not solve this mismatch. *B*_1_ homogeneity tests on a phantom (not shown) indicated that the PRESS pulse angles were correct and therefore not responsible for these effects.

TE = 32 ms prostate MRSI was impaired by considerable lipid contamination, even with the use of six REST slabs, and thus the usefulness of the approach is diminished. The quantification of lipids in the prostate could be of interest, but, as the origin of lipid resonance signals in this study was ambiguous because of the chemical shift contamination from lipid tissues lining the prostate, these results were not included. Other methods existing for lipid suppression in short-TE spectroscopy, such as conformal-voxel MRS, merit further investigation, however, in order to take full advantage of the increased visualisation of metabolite signals at this TE 36,37. Another cause of reduced metabolite visibility was a loss in signal reception from voxels distant from the endorectal coil. The reduced ability of prostate MRSI to depict anterior prostate metabolic levels is important because only 68% of prostate tumours occur in the PZ, adjacent to the endorectal coil 38.

However, our study indicates that, in lipid-uncontaminated spectra (using the CRLB cut-off criterion), TE = 32 ms MRSI is better than TE = 100 ms MRSI for the visualisation of spermine, choline, creatine and myo-inositol in the prostate (see Table 1) (citrate could not be modelled correctly at TE = 100 ms, but showed 99% visibility at TE = 32 ms). The TE = 32 ms 2D MRSI protocol lasts 6 min, with a further 2 or 3 min for positioning of the voxel and saturation bands. Currently, the MRSI data are processed offline using jMRUI for peak area analysis, followed by correction for relaxation time effects. However, for clinical application, 3D MRSI with effective lipid suppression must be performed in the prostate in order to interrogate the entire prostate. In addition, the spectral and data processing must be automated so that metabolite concentrations can be presented simply, alongside the MR images.

## CONCLUSION

With short-TE (TE = 32 ms) MRSI, absolute concentrations of citrate, spermine, choline, creatine and myo-inositol can be calculated in greater than 75% of lipid-free prostate voxels. Individual metabolite concentrations provide greater functional information than the previously used metabolite ratio [(choline + polyamine + creatine)/citrate] and could therefore be of greater use for the clinical assessment of tumours. TE = 100 ms MRSI suffers from less lipid contamination than TE = 32 ms MRSI, but has poorer metabolite visibility amongst lipid-free voxels and more *T*_2_ variation sensitivity. In the future, the value of individual metabolite concentrations visualised using MRSI with TE = 32 ms would be to help characterise otherwise equivocal regions within the prostate.

## Abbreviations

2D: two-dimensional
3D: three dimensional
CG: central gland
CRLB: Cramér–Rao lower bound
CSA: chemical shift artefact
DW MRI: diffusion-weighted MRI
FID: free induction decay
HLSVD: Hankel–Lanczos singular value decomposition
PRESS: point-resolved spectroscopy
PZ: peripheral zone
QUEST: quantitation using quantum estimation
REST: regional saturation
TI: inversion time.
